# Genetic Manipulation of Calcium Release-Activated Calcium Channel 1 Modulates the Multipotency of Human Cartilage-Derived Mesenchymal Stem Cells

**DOI:** 10.1155/2019/7510214

**Published:** 2019-02-17

**Authors:** Shuang Liu, Minae Takahashi, Takeshi Kiyoi, Kensuke Toyama, Masaki Mogi

**Affiliations:** ^1^Department of Pharmacology, Ehime University Graduate School of Medicine, Shitsugawa, Toon-shi, Ehime 791-0295, Japan; ^2^Department of Bioscience, Integrated Center for Sciences, Ehime University, Shitsukawa, Toon-shi, Ehime 791-0295, Japan

## Abstract

Calcium is a ubiquitous intracellular messenger that has a crucial role in determining the proliferation, differentiation, and functions of multipotent mesenchymal stem cells (MSCs). Our study is aimed at elucidating the influence of genetically manipulating Ca^2+^ release-activated Ca^2+^ (CRAC) channel-mediated intercellular Ca^2+^ signaling on the multipotency of MSCs. The abilities of genetically engineered MSCs, including CRAC-overexpressing and CRAC-knockout MSCs, to differentiate into multiple mesenchymal lineages, including adipogenic, osteogenic, and chondrogenic lineages, were evaluated. CRAC channel-mediated Ca^2+^ influx into these cells was regulated, and the differentiation fate of MSCs was modified. Upregulation of intracellular Ca^2+^ signals attenuated the adipogenic differentiation ability and slightly increased the osteogenic differentiation potency of MSCs, whereas downregulation of CRACM1 expression promoted chondrogenic differentiation potency. The findings demonstrated the effects of genetically manipulating MSCs by targeting CRACM1. CRAC-modified MSCs had distinct differentiation fates to adipocytes, osteoblasts, and chondrocytes. To aid in the clinical implementation of tissue engineering strategies for joint regeneration, these data may allow us to identify prospective factors for effective treatments and could maximize the therapeutic potential of MSC-based transplantation.

## 1. Introduction

Advancement in understanding the pathogenesis of joint destruction by autoimmune disorders, such as rheumatoid arthritis and systemic lupus erythematosus, has benefited the development of immunosuppressants that modulate cytokine networks and pathological immune cells. Therapeutic approaches using mesenchymal stem cells (MSCs) for autoimmune diseases are based on their immunomodulatory capabilities to achieve systemic immunosuppression and multipotent differentiation for skeletal regeneration [1]. Culture-expanded MSCs, mainly bone marrow-derived MSCs, have been tested in preclinical models and trials of inflammatory arthritis. The ability to reset the immune system by reducing deleterious Th1 and Th17 responses and enhance the protective regulatory T cell response has been demonstrated [2]. However, although studies in experimental models suggest that the migration of MSCs adjacent to the joint cavity is crucial for chondrogenesis during embryogenesis, a previous study has shown that synovium-derived MSCs might be the primary drivers of cartilage repair in adulthood [3, 4]. Therefore, our understanding of the regenerative capacity of joint-resident multipotent MSCs is still limited. For cartilage regeneration, further exploration of MSC-based joint regeneration is required.

Calcium release-activated calcium (CRAC) channels, also known as *ORAI*, have been investigated as a target for novel immunosuppressive drug development [5–8]. CRAC channels were originally identified as fundamental regulators of store-operated calcium entry (SOCE) in nonexcitable cells such as most immune cells [9–12]. CRAC channels are activated through binding of the endoplasmic reticulum Ca^2+^ sensor stromal insertion molecule to CRAC channel proteins CRACM1, CRACM2, and CRACM3, of which CRACM1 is the major pore-forming subunit of the CRAC channel in humans [13]. A recent study has shown that CRAC channels are expressed in human dental pulp-derived MSCs and modulate intracellular ATP-induced Ca^2+^ responses [14]. The importance of CRAC channels in joint biology was demonstrated by regulation of osteogenic differentiation through bone morphogenetic protein signaling in CRAC-deficient mouse bone marrow-derived MSCs [15]. Such evidence indicates that the differentiation fate of multipotent MSCs can be manipulated by modulating the intracellular Ca^2+^ signaling pathway via CRAC channels.

To test this hypothesis, the present study investigated the effect of intracellular Ca^2+^ signaling on the multipotency of human cartilage-derived MSCs. Genetic manipulation techniques were used to achieve various levels of Ca^2+^ influx into MSCs. The abilities of genetically engineered MSCs to differentiate into multiple mesenchymal lineages, including adipogenic, osteogenic, and chondrogenic lineages, were evaluated. Our study demonstrated that a cell transplantation tool for joint regeneration could be achieved by appropriate genetic manipulation of CRAC channels in MSCs.

## 2. Material and Methods

### 2.1. Cell Culture and Genetic Modulation of CRACM1

Finger cartilage-derived primary MSCs were purchased from the Japanese Collection of Research Bioresources Cell Bank (JCRB) (Japan). According to the instructions from JCRB, MSCs were cultured in preconditioning Poweredby10 medium at 37°C in a humidified atmosphere with 5% CO_2_. All experiments were performed with passage 3 MSCs. The research protocol was approved by the Ethics Committee of the Ehime University School of Medicine (approval no. 1409004).

PcDNA3.1-Orai1 carrying CRACM1 was a gift from Anjana Rao (Addgene plasmid # 21638) [16], which was transfected into MSCs using Lipofectamine® 3000 Reagent (Thermo Fisher Scientific K.K., Tokyo, Japan), according to the manufacturer's instructions. The culture medium was replaced with Poweredby10 medium containing 400 *μ*g/ml Geneticin™ Selective Antibiotic at 7 days after transfection.

For CRACM1 knockout, a genome-wide ORAI1 Human Gene Knockout Kit (OriGene Technologies Inc., Rockville, MD, USA), which is designed based on clustered regularly interspaced short palindromic repeat (CRISPR) technology, was used. According to the manufacturer's instructions, gRNA vectors and a linear EF1a-GFP-P2A-Puro donor were cotransfected into MSCs using TurboFectin 8.0 (OriGene). MSCs were collected using a 0.25% Trypsin and 1 mM EDTA solution at 7 days after transfection. GFP-positive MSCs were sorted using a BD FACSAria™ Cell Sorter (BD Biosciences, Tokyo, Japan).

Expression levels of CRACM1 mRNA were detected in wild-type MSCs, pcDNA3.1-Orai1-transfected MSCs (M1-MSCs), and CRACM1-knockout MSCs (KOM1-MSCs) by reverse transcription-polymerase chain reaction (PCR) at 14 days after transfection. Total RNA was extracted, and mRNA levels of CRACM1 and glyceraldehyde-3-phosphate dehydrogenase (GAPDH) were detected using a Takara RNA PCR kit (AMV) Ver. 3.0 (Takara Bio, Shiga, Japan), according to the manufacturer's instructions. The specific oligonucleotide primers used for amplification were as follows: GAPDH (225 bp): forward, 5′-AAGGTCGGAGTCAACGGATT-3′ and reverse, 5′-CTCCTGGAAGATGGTGATGG-3′ and CRACM1 (578 bp): forward, 5′-TCGGTCAAGGAGTCCCCCAT-3′ and reverse, 5′-GTCCTGAAGCGGGAACTC-3′. The elative expression of CRACM1 and GAPDH mRNAs was assessed according to the manufacturer's instructions using the One Step SYBR PrimeScript PLUS RT-PCR Kit (Takara Bio Inc., Shiga, Japan). Fluorescence emissions of the probes were monitored and analyzed using an Applied Biosystems 7500 Fast Real-Time PCR system (Thermo Fisher Scientific K.K., Tokyo, Japan). Specific oligonucleotide primers were designed according to published sequences as follows: CRACM1 forward primer and AGCCTCAACGAAGCATCCCAT; CRACM1 reverse primer: CTGATCATGAGCGCAAACAGG and GAPDH forward primer, TGAGTACGTCGTGGAGTTCCACTG and GAPDH reverse primer, CACCACCAACTGCTTAGCACC. Relative quantification of gene expression was performed by the comparative Ct method.

### 2.2. Ca^2+^ Imaging

MSCs, M1-MSCs, and KOMSCs were seeded on an imaging dish at the center of a coverslip. Before imaging, the cells were incubated in a loading buffer for 1 h at 37°C. The loading buffer consisted of 1 mg/ml bovine serum albumin (BSA), Fluo-4, AM (1×) in dimethyl sulfoxide, and PowerLoad™ concentrate (1×) (Thermo Fisher Scientific) in Hank's buffered salt solution (HBSS, Ca^2+^+; 13.7 mM NaCl, 0.54 mM KCl, 0.05 mM MgCl_2_-6H_2_O, 0.04 mM MgSO_4_-7H_2_O, 0.04 mM KH_2_PO_4_, 0.03 mM Na_2_HPO_4_-7H_2_O, 1.3 mM CaCl_2_, 5.5 mM glucose, and 4.2 mM NaHCO_3_). Then, the culture medium was replaced with Ca^2+^-free HBSS. After Fluo-4 loading, the cells were observed under an all-in-one fluorescence microscope (BZ-X700; Keyence, Tokyo, Japan) at ×400 magnification. Image acquirement was performed at room temperature. The imaging period was 200 s without stimulation, followed by 500 s after stimulation. After the 200 s baseline measurement, the cells were slowly perfused with a thapsigargin (TG) solution (Ca^2+^ free) at a final concentration of 0.5 *μ*M. The cells were then perfused with a CaCl_2_ solution (2 mM) at the 400 s time point. All acquired images were saved as AVI files, and the average fluorescence intensity of each cell was analyzed by ImageJ according to established protocols.

### 2.3. Identification of Multipotency in MSCs

A Human Mesenchymal Stem Cell Functional Identification Kit (R&D Systems, Minneapolis, MN, USA) was used to induce and detect the ability for differentiation into multiple mesenchymal lineages.

For adipogenic differentiation, MSCs were seeded on a 96-well imaging plate and cultured in basic Poweredby10 medium until 100% confluent. Then, the medium was replaced with adipogenic differentiation medium containing adipogenic supplements including hydrocortisone, isobutylmethylxanthine, and indomethacin. Seven days later, the MSCs were fixed with 4% paraformaldehyde for 20 min at room temperature and blocked in 0.3% Triton X-100, 1% BSA, and 10% donkey serum in phosphate-buffered saline. The cells were stained using a primary anti-mouse fatty acid binding protein (mFABP) antibody, followed by an anti-goat secondary antibody. After washing, the cells were counterstained with Hoechst® 33342 (0.5 *μ*g/ml) in distilled water and visualized using a fluorescence microscope (BZ-9000; Keyence). For fluorescence quantification, image acquirement was performed in the imaging chamber using MetaXpress software (Molecular Devices, Tokyo, Japan) at room temperature [17]. Thirty-two fields were captured in each well with 100–400 ms exposure times at a magnification of ×200. All images were 16 bit. The cells were identified using the Transfluor module of MetaXpress software, and the integrated intensity of each image was determined under the background-subtracted condition. For functional analysis, lipid droplets, which were formed during adipogenic differentiation, were stained in fixed cells using an Oil Red O stain kit (Abcam, Tokyo, Japan), according to the manufacturer's instruction.

For osteogenic induction, MSCs seeded on a 96-well imaging plate were cultured in osteogenic supplement-containing medium for 21 days. The osteogenic supplements were dexamethasone, ascorbate phosphate, and *β*-glycerophosphate. The induced osteocytes were labeled using primary anti-human osteocalcin and secondary anti-mouse antibodies. The image-capturing methods were similar to those for adipogenic detection. The activity of alkaline phosphatase (ALP) was assessed by staining fixed cells undergoing osteogenic differentiation on day 14 using an ALP staining kit (Wako, Tokyo, Japan), according to the manufacturer's protocol.

MSCs (2.5 × 10^5^ cells) were collected from culture plates and pelleted for chondrogenic differentiation. Chondrogenic supplements dexamethasone, ascorbate phosphate, proline, pyruvate, and recombinant tumor growth factor-*β*3 and ITS supplement including insulin, transferrin, selenious acid, BSA, and linoleic acid were added to the basal MSC culture medium. After 21 days, fixed chondrocyte pellets were embedded in OCT compound and sectioned on a cryotome. After blocking and permeabilization using 0.3% Triton X-100, 1% BSA, and 10% donkey serum in phosphate-buffered saline, frozen sections were incubated with a primary anti-human aggrecan antibody or anti-human CD44 antibody and then a secondary antibody. The sections were mounted using ProLong™ Gold Antifade Mountant with 4′,6-diamidino-2-phenylindole (DAPI). Images were captured using a fluorescence microscope. The positive area was segmented from the background image, and integrated intensity was quantified using ImageJ. The total area was segmented by a greyscale. More than four fields and the average results of four sections per sample were used for semiquantitative analysis. The integrated intensity was divided by the total area to determine the relative integrated intensity per mm^2^.

### 2.4. Statistical Analysis

All experiments were designed in a completely randomized multifactorial format. The sample distributions were analyzed using the Kolmogorov-Smirnov test. Pairwise comparisons were performed using the two-tailed Student *t*-test. *P* < 0.05 was considered as significant. Data were analyzed with GraphPad Prism 7.01 (GraphPad Software, La Jolla, CA, USA).

## 3. Results

### 3.1. Modulation of SOCE by Genetically Engineering CRACM1 in MSCs

**Figure 1:**
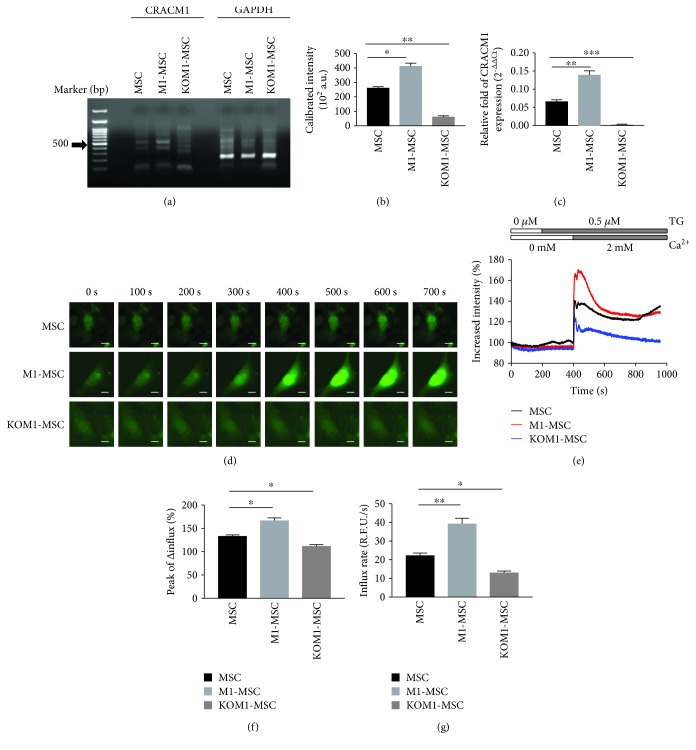
Modulation of Ca^2+^ in CRAC-manipulated MSCs. The following experiments were conducted at 7 days after gene transfection of wild-type MSCs, pcDNA3.1-Orai1-transfected MSCs (M1-MSCs), and CRACM1-specific gRNA vector and linear EF1a-GFP-P2A-Puro donor-cotransfected MSCs (KOM1-MSCs). (a) PCR amplification of reverse transcription products produced the expected band following genetic modification. Molecular marker (lane 1); CARCM1 expression (523 bp) in MSCs, M1-MSCs, and KOM1-MSCs (lanes 3, 4, and 5, respectively); and GAPDH expression (214 bp) in MSCs, M1-MSCs, and KOM1-MSCs (lanes 7, 8, and 9, respectively) are shown. (b) CRACM1 mRNA expression in MSCs, M1-MSCs, and KOM1-MSCs (a.u. (arbitrary units); ^∗^*P* < 0.05 and ^∗∗∗^*P* < 0.001). Results are expressed as mean ± SEM (*n* = 4). (c) The relative expression of CRACM1 to housekeeping GAPDH in MSCs, M1-MSCs, and KOM1-MSCs using quantitative real-time PCR. Relative fold of CRACM1 expression was achieved using the comparative Ct method (2^-ΔΔCt^) (^∗∗^*P* < 0.01 and ^∗∗∗^*P* < 0.001). (d) Time sequential patterns of Ca^2+^ imaging in single MSCs, M1-MSCs, and KOM1-MSCs. The imaging period was 200 s without stimulation, followed by 500 s after stimulation. After a 200 s baseline measurement, cells were slowly perfused with TG (0.5 *μ*M) and then perfused with a CaCl_2_ solution (2 mM) at the 400 s time point (scale bar: 10 *μ*m). (e) Typical Ca^2+^ influx patterns of MSCs, M1-MSCs, and KOM1-MSCs shown in (c). (f) Maximum increases in fluorescent intensity values of MSCs, M1-MSCs, and KOM1-MSCs. Quantification was performed using images acquired from 100–120 cells of each group (^∗^*P* < 0.05). Results are expressed as mean ± SEM. (g) Initial rate of Ca^2+^ influx (in the first 15 s after Ca^2+^ addition) into MSCs, M1-MSCs, and KOM1-MSCs. Quantification was performed using images acquired from 100–120 cells of each group (^∗^*P* < 0.05 and ^∗∗^*P* < 0.01). Results are expressed as mean ± SEM.

To modulate SOCE in MSCs, CRACM1 expression on the plasma membrane, which is a pore-forming unit of the channel, was manipulated by genetic modification. CRACM1 mRNA expression was evaluated in wild-type MSCs, M1-MSCs, and KOM1-MSCs (Figures 1(a) and 1(b)). Compared with MSCs, the CRACM1 mRNA expression level was enhanced in M1-MSCs, whereas its expression was absent in KOM1-MSCs in which CRACM1 was genetically knocked out by the CRISPR/CRISPR-associated protein technique. The results of quantitative real-time PCR supported the data obtained from gel analysis (Figure 1(c)).

The modification of CRACM1 expression directly influenced SOCE in MSCs, according to the results of Ca^2+^ imaging in single cells (Figures 1(d)–1(g)). Typical Ca^2+^ influx images at the indicated time points are shown in Figure 1(d). Time-dependent patterns of intracellular Ca^2+^ in these cells were quantified (Figure 1(e)). Passive depletion of Ca^2+^ stores and activation of CRAC channels in MSCs were induced by TG, a sarco-/endoplasmic reticulum Ca^2+^-ATPase pump inhibitor. Elevation of intracellular Ca^2+^, reflected by the increase in relative fluorescence intensity, was observed in TG-stimulated MSCs, M1-MSCs, and KOM1-MSCs upon addition of extracellular Ca^2+^. According to the maximum increase in fluorescent intensity values obtained from 100–200 cells of each group, M1-MSCs had higher influx peaks (*P* = 0.027) and rates (*P* = 0.009) of Ca^2+^ influx compared with M1-MSCs (Figures 1(f) and 1(g)). In addition, although genetic knockout of CRACM1 did not completely attenuate SOCE, a significant decrease in Ca^2+^ influx was observed in KOM1-MSCs with a 16.90% decrease in the influx peak and 45.84% decrease in the influx rate.

Taken together, SOCE was successfully regulated by genetic manipulation of CRACM1 in MSCs.

### 3.2. Upregulation of CRACM1 Inhibits the Adipogenic Differentiation Potential of MSCs

**Figure 2:**
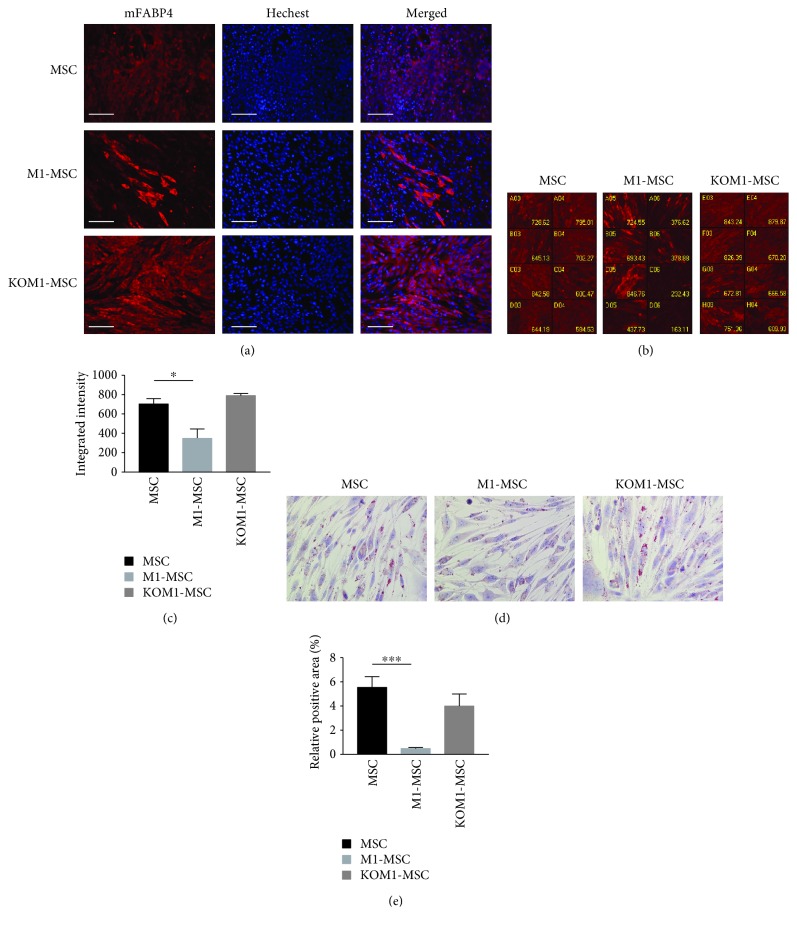
Overexpression of CRACM1 inhibits MSC differentiation to adipocytes. MSCs, M1-MSCs, and KOM1-MSCs were cultured in adipogenic differentiation medium for 7 days. Cells were then stained for mFABP (red) as a marker of adipocytes and counterstained with Hoechst® 33342 for nuclear staining (blue). (a) Typical images of MSCs, M1-MSCs, and KOM1-MSCs observed by fluorescence microscopy (×200; scale bar: 50 *μ*m). (b) Typical imaging screening panel for quantification of mFABP4 expression. MSCs, M1-MSCs, and KOM1-MSCs were seeded on 8-well plates, and 32 fields were captured in each well using a high-throughput image quantitation system. One of 32 fields is shown. Well number and average intensity are indicated on the image. (c) FABP expression in MSCs, M1-MSCs, and KOM1-MSCs. The average fluorescent intensity was obtained from 256 images for each group (^∗^*P* < 0.05). Results are expressed as mean ± SEM. (d) Typical images of lipid droplets analysis. Lipid droplets, which were stained using Oil Red O, present as bright refractive round structures. (×400; scale bar: 25 *μ*m). (e) Relative positive area of lipid droplet analysis. The red-stained area was segmented from the background, and the relative positive area was quantified. More than four fields per section and an average of five sections from each sample were used for semiquantitative analysis (^∗∗∗^*P* < 0.001). Results are expressed as mean ± SEM.

Multipotency was evaluated in CRACM1-manipulated MSCs. The adipogenic differentiation potential was evaluated using FABP4, commonly known as adipocyte protein 2, which has been extensively used as a marker for differentiated adipocytes. Typical images of immunofluorescence staining are shown in Figure 2(a). FABP4 expression was increased in KOM1-MSCs, whereas induction of FABP4 expression was obviously inhibited in CRAC-overexpressing M1-MSCs compared with wild-type MSCs. A high-throughput imaging screening system was used for FABP4 quantification (Figures 2(b) and 2(c)). A typical screening panel of 32 panels is shown (Figure 2(b)). After calibration by the nuclear number, the average intensity of the FABP-4-positive area in M1-MSCs was decreased to 49.53% (*P* = 0.011) of that in MSCs (Figure 2(c)). The increase in FABP4 expression in KOM1-MSCs was not significant (*P* = 0.143). A significant decrease in lipid droplet formation was observed in M1-MSCs compared with wild-type MSCs (*P* < 0.001), which supported the results of FABP4 detection (Figures 2(d) and 2(e)). These results suggested that increased SOCE inhibited adipogenic differentiation of MSCs.

### 3.3. Overexpression of CRACM1 Induces Osteogenic Differentiation

**Figure 3:**
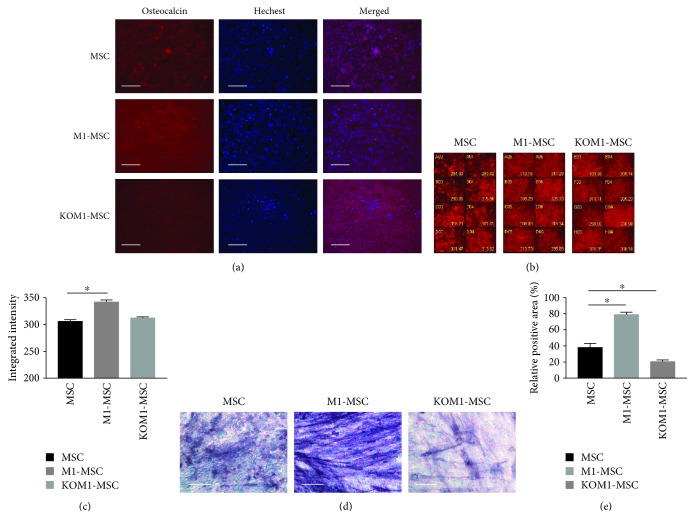
Osteogenic differentiation potencies of MSCs, M1-MSCs, and KOM1-MSCs. Osteogenic differentiation potencies of MSCs, M1-MSCs, and KOM1-MSCs were observed after 14 days of induction in osteogenic medium. Osteocalcin (red) was used as a marker of osteoblasts, and nuclei were stained with Hoechst (blue). (a) Typical images of MSCs, M1-MSCs, and KOM1-MSCs obtained by fluorescence microscopy (×200; scale bar: 50 *μ*m). (b) Typical imaging screening panel for quantification of osteocalcin expression. Well number and average intensity are indicated on the image. (c) Osteocalcin expression in MSCs, M1-MSCs, and KOM1-MSCs. The average fluorescent intensity was obtained from 256 images for each group (^∗^*P* < 0.05). Results are expressed as mean ± SEM. (d) Typical images of alkaline phosphatase analysis (×400; scale bar: 25 *μ*m). (e) Relative positive area of alkaline phosphatase staining. The purple-blue-stained area was segmented from the background, and the relative positive area was quantified. More than four fields per section and an average of five sections from each sample were used for semiquantitative analysis (^∗^*P* < 0.05). Results are expressed as mean ± SEM.

Compared with the adipogenic differentiation potential, osteogenic differentiation of MSCs, M1-MSCs, and KOM1-MSCs exhibited different induction patterns. The osteogenic differentiation potential was maintained in these cells with more than 90% committing to this lineage, according to the expression of osteocalcin, a marker of differentiated osteoblasts (Figure 3). When an organic scaffold is enriched with osteocalcin, matrix mineralization occurs [18]. Under the influence of dexamethasone, ascorbate phosphate, and *β*-glycerophosphate, MSCs formed aggregates or nodules and increased their osteocalcin expression (Figures 3(a)–3(c)). Quantitative analysis revealed that the levels of osteocalcin in MSCs, and KO-MSCs, were similar but increased in M1-MSCs compared with MSCs (*P* = 0.047). ALP activity in M1-MSCs was increased significantly compared with that in wild-type MSCs (*P* = 0.012), but it was suppressed in KOM1-MSCs (*P* = 0.044) (Figures 3(d) and 3(e)). Based on these results, upregulation of intracellular Ca^2+^ signals in MSCs may enhance the osteogenic differentiation potential and benefit bone regeneration.

### 3.4. Distinct Contribution of SOCE to Chondrogenic Differentiation

**Figure 4:**
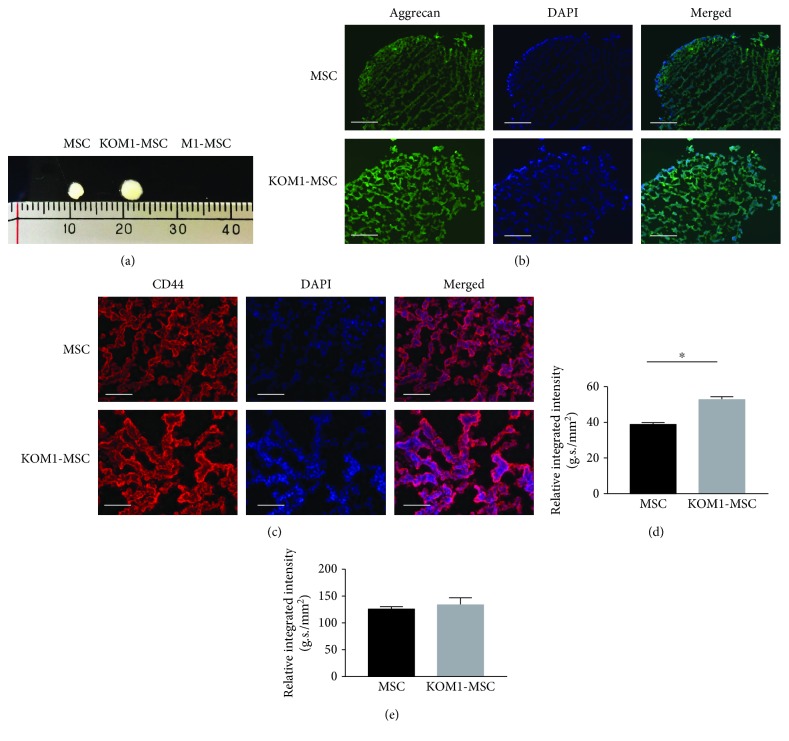
Chondrogenic differentiation potencies of MSCs, M1-MSCs, and KOM1-MSCs. (a) Micromass formation by MSCs, M1-MSCs, and KOM1-MSCs after 21 days of induction culture. M1-MSCs failed to form any micromasses. (b) Typical images of MSCs, M1-MSCs, and KOM1-MSCs obtained by fluorescence microscopy (×200; scale bar: 50 *μ*m). Fixed chondrocyte pellets were embedded in OCT compound and sectioned on a cryotome. The sections were stained with an anti-human aggrecan antibody (green) and DAPI (blue). (c) Typical images of CD44 expression in MSCs and KOM1-MSCs. The sections were stained with an anti-human CD44 antibody (red) and DAPI (blue) (×400; scale bar: 25 *μ*m). (d) Quantification of aggrecan expression in micromasses formed by MSCs, M1-MSCs, and KOM1-MSCs. More than four fields and average results of four sections for per sample were used for semiquantitative analysis. The integrated intensity was divided by the total area to determine the relative integrated intensity per mm^2^ (^∗^*P* < 0.05). Results are expressed as mean ± SEM. (e) Quantification of CD44 expression in the micromasses formed by MSCs and KOM1-MSCs. Results are expressed as mean ± SEM.

To promote chondrogenic differentiation, MSCs were gently centrifuged and a pelleted micromass was formed after 21 days of culture (Figure 4(a)). CRACM1-overexpressing M1-MSCs completely lost their ability to differentiate to the chondrogenic lineage, and only MSCs and KOM1-MSCs formed detectable micromasses. Micromasses formed by MSCs and KOM1-MSCs developed a multilayered matrix-rich morphology, and immunohistological analysis showed CD44-positive cellular aggregation and an aggrecan-rich extracellular matrix (Figures 4(b) and 4(c)). Quantification of the aggrecan fluorescent intensity suggested a stronger chondrogenic differentiation ability in KOM1-MSCs than in MSCs (*P* = 0.031) (Figure 4(c)). There was no significant difference in the expression of CD44 between MSCs and KOM1-MSCs (Figure 4(e)). Therefore, genetically engineering MSCs promoted their cartilage formation ability via modulation of CRAC channels.

## 4. Discussion

In the present study, we analyzed the multipotency of CRACM1-manipulated human cartilage-derived MSCs. CRAC channel-mediated Ca^2+^ influx into these cells was regulated, and the differentiation fates of multipotent MSCs were modified. Upregulation of intracellular Ca^2+^ signals attenuated the adipogenic differentiation ability and slightly increased the osteogenic differentiation potency of MSCs, whereas downregulation of CRACM1 expression promoted chondrogenic differentiation potency. For joint regeneration, these genetically engineered MSCs could be chosen according to the particular tissue-reforming purpose.

Ca^2+^ channels function as a gateway for extracellular Ca^2+^ diffusion across lipid membrane barriers of MSCs, while the modulatory mechanism and functions of intracellular Ca^2+^ signaling in MSCs have not been elucidated conclusively yet. A previous study demonstrated the presence of stretch-activated calcium channels, which are required for mechanotransduction of MSCs, and voltage-gated calcium channels that are activated by membrane depolarization and mediate Ca^2+^ influx [19]. Activation of A transient receptor potential canonical channel 1, which forms nonselective cation channels, is generally linked to stimulation of plasma membrane receptors coupled to PLC*γ* [20] and has been reported in dopaminergic differentiated MSCs [21]. Although the expression and functional statuses of this channel in multipotent MSCs are unknown, it is a possible component of SOCE mechanisms upon depletion of intracellular Ca^2+^ stores. The CRAC channel was recently identified as an intracellular Ca^2+^ signal modulator in MSCs [14]. The composition and function of CRAC have been carefully dissected in immune cells. As novel molecular targets for immunosuppressant development, a series of cellular targets for CRAC-inhibitors, including B lymphocytes, T lymphocytes, and osteoclasts, have shown great therapeutic potential, which have been highlighted. Specific immune modulators with improved efficacy have been developed for the management of autoimmune diseases. However, CRAC is so fundamental to nonexcitable cell physiology that loss of CRACM1 causes severe combined immunodeficiency in humans and poor survival rates of gene-trapped mice [12]. It has been a challenge to genetically manipulate CRACM1 in immune cells. In the present study, we successfully manipulated the expression of CRACM1 in MSCs [22]. There were no obvious differences in cell homeostasis or proliferation rates among MSCs, M1-MSCs, and KOM1-MSCs. Genetic knockout of CRACM1 did not completely attenuate SOCE in MSCs. These results suggest that, although CRAC channels regulate the SOCE ability and contribute to the determination of differentiation fate, the initial Ca^2+^ modulator in MSCs may be limited not only to CRAC channels but also to other candidates that support basic SOCE and maintain intracellular Ca^2+^ hemostasis, which should also be elucidated.

Among several joint tissue-repairing and tissue engineering approaches, the high proliferative capacity of cultured MSCs and their osteogenic and chondrogenic capabilities have catapulted them to the forefront of cell-based therapy for joint-destructive diseases. Bone marrow-derived MSCs have been applied to joint repair strategies that employ combinations of culture-expanded cells and adjuncts including scaffolds and pharmaceutical agents [23]. A clinical trial has demonstrated that autologous MSC transplantation is an effective approach to promote the repair of articular cartilage defects [24]. Autologous joint tissue-derived MSCs would be more advantageous than allogeneic bone marrow-derived MSCs in terms of a higher cartilage formative capacity and lower risk of adverse clinical responses for repeated intra-articular application [25]. The tissue resource of cartilage-derived MSCs in our future studies could be articular cartilage explants obtained from patients who have undergone keen joint prosthetic replacement arthroplasty for therapeutic purposes. However, the biology of cartilage-resident MSCs has not been fully elucidated yet [3]. A study by Archer's group on the morphology of a mammalian joint indicated that chondrocytes are likely replenished from the superficial zone rather than from the deep zone [26]. There are two subpopulations of human cartilage-resident MSC-like progenitor cells, which can be separated by their proliferative potential and capacity for telomere maintenance [27]. The cartilage-derived MSCs used in the present study showed limited self-renewal and colony-forming abilities, and their multipotency for adipogenic, osteogenic, and chondrogenic differentiation was demonstrated.

Ca^2+^ influx is critical for osteogenesis and chondrogenesis of human MSCs [28, 29]. High extracellular Ca^2+^ has been shown to enhance osteogenesis and osteoblasts that are known to propagate Ca^2+^ signals. Ca^2+^ influx via the plasma membrane is required for chondrogenesis of high-density chicken MSC cultures [30]. Although CRAC may not be the only mechanism to modulate SOCE, modifying the expression of CRACM1 results in up- or downregulation of Ca^2+^ influx, and responsiveness of intracellular Ca^2+^ is therefore adjusted. According to our results, CRAC-downregulated MSCs could differentiate to chondrocytes and form a micromass. These cells could be useful for cell-based in situ transplantation with proper scaffolds or growth factors, which can integrate cells with adjacent cartilage. KOM1-MSCs may promote cartilage reformation, and M1-MSCs could be used for treatment of bone erosion diseases. The pathogenesis of joint-destructive diseases is complex and heterogeneous, in which both disease initiation and progression depend on multiple joint structures including the cartilage, bone, ligaments, meniscus, and synovium. Genetically engineered MSCs could be used for strategy modification to achieve maximum therapeutic efficiency of MSC-based transplantation. In our planned future study, these genetically manipulated MSC will be transplanted into the site of destructed joints, and their feasibility and efficiency for joint regeneration will be evaluated.

In addition, native joint-resident MSCs, such as cartilage-resident MSCs used in the present study, have endogenous reparative capabilities [3]. According to the management priorities, the function of CRAC channels in joint-residue MSCs could be regulated via pharmacological agents or gene modification. In our previous study, intra-articular gene silencing of CRACM1 efficiently suppressed joint destruction in a collagen-induced arthritis murine model [31]. The inhibition of cartilage destruction in CRACM1-downregulated joints may be due to not only direct functional inhibition of infiltrated immunological cells but also enhancement of endogenous cartilage-reparative capabilities. Therefore, modulation of osteogenic and chondrogenic capabilities in ex vivo culture-expanded MSCs or native joint-resident MSCs may optimize cell-based repair strategies to reestablish joint homeostasis.

## 5. Conclusion

In conclusion, the present study demonstrated the effects of genetically manipulating MSCs by targeting CRACM1. CRAC-modified MSCs had distinct differentiation fates to adipocytes, osteoblasts, and chondrocytes. To aid the clinical implementation of tissue engineering strategies for joint regeneration, the evidence provided here may allow us to identify prospective factors for effective treatment and could maximize the therapeutic potential of MSC-based transplantation.

## Data Availability

All data generated or analyzed during this study are included in this published article. Readers may access the data underlying the findings of this study by contacting the corresponding author, Shuang Liu, at liussmzk@m.ehime-u.ac.jp.

## References

[1] El-Jawhari J. J., El-Sherbiny Y. M., Jones E. A., McGonagle D. (2014). Mesenchymal stem cells, autoimmunity and rheumatoid arthritis. *QJM*.

[2] MacDonald G. I. A., Augello A., de Bari C. (2011). Role of mesenchymal stem cells in reestablishing immunologic tolerance in autoimmune rheumatic diseases. *Arthritis & Rheumatism*.

[3] McGonagle D., Baboolal T. G., Jones E. (2017). Native joint-resident mesenchymal stem cells for cartilage repair in osteoarthritis. *Nature Reviews Rheumatology*.

[4] Baboolal T. G., Mastbergen S. C., Jones E., Calder S. J., Lafeber F. P. J. G., McGonagle D. (2016). Synovial fluid hyaluronan mediates MSC attachment to cartilage, a potential novel mechanism contributing to cartilage repair in osteoarthritis using knee joint distraction. *Annals of the Rheumatic Diseases*.

[5] Lin F. F., Elliott R., Colombero A. (2013). Generation and characterization of fully human monoclonal antibodies against human Orai1 for autoimmune disease. *Journal of Pharmacology and Experimental Therapeutics*.

[6] Sadaghiani A. M., Lee S. M., Odegaard J. I. (2014). Identification of Orai1 channel inhibitors by using minimal functional domains to screen small molecule microarrays. *Chemistry & Biology*.

[7] Liu S., Hasegawa H., Takemasa E. (2017). Efficiency and safety of CRAC inhibitors in human rheumatoid arthritis xenograft models. *The Journal of Immunology*.

[8] Miyoshi M., Liu S., Morizane A. (2018). Efficacy of constant long-term delivery of YM-58483 for the treatment of rheumatoid arthritis. *European Journal of Pharmacology*.

[9] Vaeth M., Zee I., Concepcion A. R. (2015). Ca^2+^ signaling but not store-operated Ca^2+^ entry is required for the function of macrophages and dendritic cells. *The Journal of Immunology*.

[10] Oh-hora M. (2009). Calcium signaling in the development and function of T-lineage cells. *Immunological Reviews*.

[11] Feske S., Prakriya M., Rao A., Lewis R. S. (2005). A severe defect in CRAC Ca^2+^ channel activation and altered K^+^ channel gating in T cells from immunodeficient patients. *The Journal of Experimental Medicine*.

[12] Vig M., Peinelt C., Beck A. (2006). CRACM1 is a plasma membrane protein essential for store-operated Ca^2+^ entry. *Science*.

[13] Liu S., Sahid M. N. A., Takemasa E. (2016). CRACM3 regulates the stability of non-excitable exocytotic vesicle fusion pores in a Ca^2+^-independent manner via molecular interaction with syntaxin4. *Scientific Reports*.

[14] Peng H., Hao Y., Mousawi F. (2016). Purinergic and store-operated Ca^2+^ signaling mechanisms in mesenchymal stem cells and their roles in ATP-induced stimulation of cell migration. *Stem Cells*.

[15] Lee S. H., Park Y., Song M. (2016). Orai1 mediates osteogenic differentiation via BMP signaling pathway in bone marrow mesenchymal stem cells. *Biochemical and Biophysical Research Communications*.

[16] Gwack Y., Srikanth S., Feske S. (2007). Biochemical and functional characterization of Orai proteins. *Journal of Biological Chemistry*.

[17] Kiyoi T., Liu S., Sahid M. N. A., Shudou M., Maeyama K., Mogi M. (2018). High-throughput screening system for dynamic monitoring of exocytotic vesicle trafficking in mast cells. *PLoS One*.

[18] Rutkovskiy A., Stensløkken K. O., Vaage I. J. (2016). Osteoblast differentiation at a glance. *Medical Science Monitor Basic Research*.

[19] Steward A. J., Kelly D. J., Wagner D. R. (2014). The role of calcium signalling in the chondrogenic response of mesenchymal stem cells to hydrostatic pressure. *European Cells and Materials*.

[20] Feske S., Skolnik E. Y., Prakriya M. (2012). Ion channels and transporters in lymphocyte function and immunity. *Nature Reviews Immunology*.

[21] Sun Y., Selvaraj S., Pandey S. (2018). MPP^+^ decreases store-operated calcium entry and TRPC1 expression in mesenchymal stem cell derived dopaminergic neurons. *Scientific Reports*.

[22] Liu S., Kiyoi T., Takemasa E., Maeyama K. (2014). Systemic lentivirus-mediated delivery of short hairpin RNA targeting calcium release-activated calcium channel 3 as gene therapy for collagen-induced arthritis. *The Journal of Immunology*.

[23] Wakitani S., Imoto K., Yamamoto T., Saito M., Murata N., Yoneda M. (2002). Human autologous culture expanded bone marrow mesenchymal cell transplantation for repair of cartilage defects in osteoarthritic knees. *Osteoarthritis and Cartilage*.

[24] Wakitani S., Nawata M., Tensho K., Okabe T., Machida H., Ohgushi H. (2007). Repair of articular cartilage defects in the patello-femoral joint with autologous bone marrow mesenchymal cell transplantation: three case reports involving nine defects in five knees. *Journal of Tissue Engineering and Regenerative Medicine*.

[25] Joswig A. J., Mitchell A., Cummings K. J. (2017). Repeated intra-articular injection of allogeneic mesenchymal stem cells causes an adverse response compared to autologous cells in the equine model. *Stem Cell Research & Therapy*.

[26] Hayes A. J., MacPherson S., Morrison H., Dowthwaite G., Archer C. W. (2001). The development of articular cartilage: evidence for an appositional growth mechanism. *Anatomy and Embryology*.

[27] Fellows C. R., Williams R., Davies I. R. (2017). Characterisation of a divergent progenitor cell sub-populations in human osteoarthritic cartilage: the role of telomere erosion and replicative senescence. *Scientific Reports*.

[28] Barradas A. M. C., Fernandes H. A. M., Groen N. (2012). A calcium-induced signaling cascade leading to osteogenic differentiation of human bone marrow-derived mesenchymal stromal cells. *Biomaterials*.

[29] Catterall W. A. (2011). Voltage-gated calcium channels. *Cold Spring Harbor Perspectives in Biology*.

[30] Fodor J., Matta C., Oláh T. (2013). Store-operated calcium entry and calcium influx *via* voltage-operated calcium channels regulate intracellular calcium oscillations in chondrogenic cells. *Cell Calcium*.

[31] Liu S., Kiyoi T., Takemasa E., Maeyama K. (2017). Intra-articular lentivirus-mediated gene therapy targeting CRACM1 for the treatment of collagen-induced arthritis. *Journal of Pharmacological Sciences*.

